# Observation of deformation twinning and martensitic transformation during nanoindentation of a transformation-induced plasticity steel

**DOI:** 10.1038/s41598-017-17824-x

**Published:** 2017-12-12

**Authors:** Zhiping Xiong, Gilberto Casillas, Ahmed A. Saleh, Shaogang Cui, Elena V. Pereloma

**Affiliations:** 1 School of Mechanical, Materials and Mechatronic Engineering, University of Wollongong, Wollongong, NSW 2522 Australia; 2 Electron Microscopy Centre, University of Wollongong, Wollongong, NSW 2519 Australia

## Abstract

For the first time, deformation twinning and martensitic transformation were observed in retained austenite in a low-alloyed transformation-induced plasticity steel using nanoindentation in conjunction with electron backscattering diffraction and transmission electron microscopy. Dislocation glide, martensite formation and deformation twinning were correlated to pop-ins and deviation from linearity in the load-displacement curve. Deformation twinning was found to enhance the stability of retained austenite. This observation furthers our understanding of RA stability during straining of low-alloyed multiphase TRIP steel.

## Introduction

Low-alloyed transformation-induced plasticity (TRIP) steel consists of polygonal ferrite, bainitic ferrite, granular bainite, retained austenite (RA) and a possible small fraction of martensite^1–3^. It combines high strength and good formability due to the transformation of RA to martensite upon straining (TRIP effect)^3,4^. Therefore, the stability of RA during deformation plays an important role in the mechanical behaviour, which has been extensively investigated^5–10^. The RA stability is dictated by many factors, among which are the grain size^6,10^, carbon content^7^, morphology^8^ and neighbouring phases^9^. Many advanced techniques have been applied to investigate the RA behaviour during straining, such as nanoindentation^11,12^, synchrotron^10,13^ or neutron^14^ diffraction and step-wise tensile testing correlated with electron backscattering diffraction (EBSD)^6^. It was demonstrated that gradual transformation of RA to martensite during straining results in the best combination of mechanical properties^5^. While deformation twinning has been rarely observed along with martensitic transformation in low-alloyed TRIP steels^15,16^, the present study reports on their concurrent formation and the effect of deformation twinning on RA stability, by correlating nanoindentation with EBSD and transmission electron microscopy (TEM). In addition, dislocation glide, deformation twinning and martensite transformation were linked to the corresponding features in the load-displacement curve, in the form of pop-ins and deviation from linearity.

## Material and Methods

**Figure 1:**
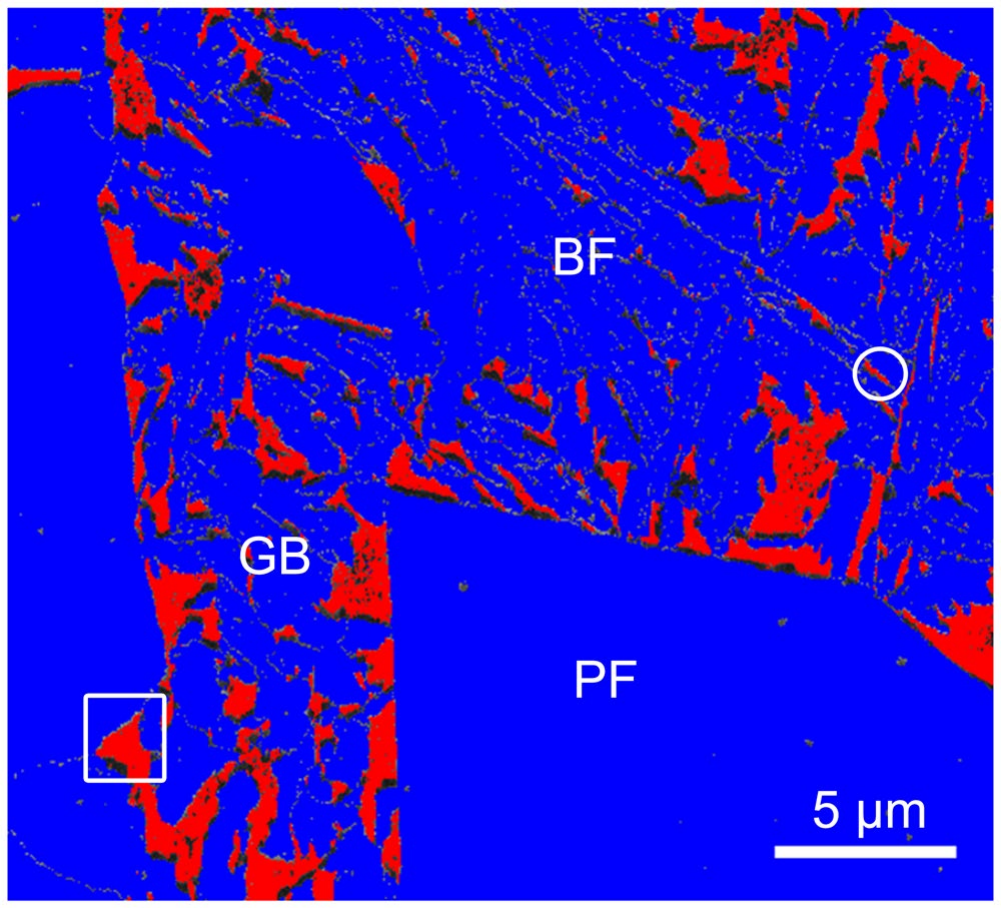
Representative phase map where red is retained austenite and blue is ferrite. *PF* is polygonal ferrite, *GB* is ferrite in granular bainite and *BF* is bainitic ferrite lath. (For interpretation of the references to colour in this figure legend, the reader is referred to the web version of this article).

A Fe-0.172 C-1.520 Si-1.610 Mn-0.0266 Al-0.0153 Cu-0.195 Cr (all compositions are given in wt. %) low-alloyed TRIP steel was produced using laboratory simulated strip casting^2^. The microstructure consisted of 55 ± 6% polygonal ferrite, 4.5 ± 0.3% RA (with an average carbon content of 1.23 ± 0.01 wt. %; measured by X-ray diffraction), and a mixture of carbide-free bainitic ferrite and granular bainite with some traces of martensite^2^.The sample for EBSD mapping and nanoindentation was mechanically polished and then electro-polished using an electrolyte of 330 ml methanol, 330 ml butoxyethanol and 40 ml perchloric acid at 50 V, ~ 1.0 mA and 17 °C for ~ 90 s. EBSD was undertaken using a JEOL JSM-7001F field emission gun – scanning electron microscope operating at an accelerating voltage of 15 kV, a probe current of ~5.1 nA, a working distance of 12 mm and a step size of 0.04 μm. The EBSD map in Fig. 1 was used for phase identification prior to nanoindentation. The ultra-microindentation system (UMIS) with a Berkovich diamond indenter was employed for nanoindentation. The peak load was chosen as 2 mN in order to study the deformation behaviour of RA as its stability in our steel is relatively high due to its high carbon content^2,7^. The load control was set to square root mode, including a gradual increase to the maximum value of 2 mN followed by unloading. Load and displacement data were recorded with a resolution of 75 nN and 0.05 nm, respectively. It is noted that the effect of grain boundaries and neighbouring phase on nanoindentation cannot be avoided in the present study due to the small RA grain size^17^. Following nanoindentation, a focused ion beam instrument (FEI Helios NanoLab G3 CX dual-beam) was used to cut lamellae from indented areas corresponding to the white square and circle in Fig. 1. The microstructure under the indentations was characterised using a JEOL JEM-ARM200F TEM operating at 200 kV.

### Data availability

The datasets generated during and/or analysed during the current study are available from the corresponding author on reasonable request.

## Results and Discussion

**Figure 2:**
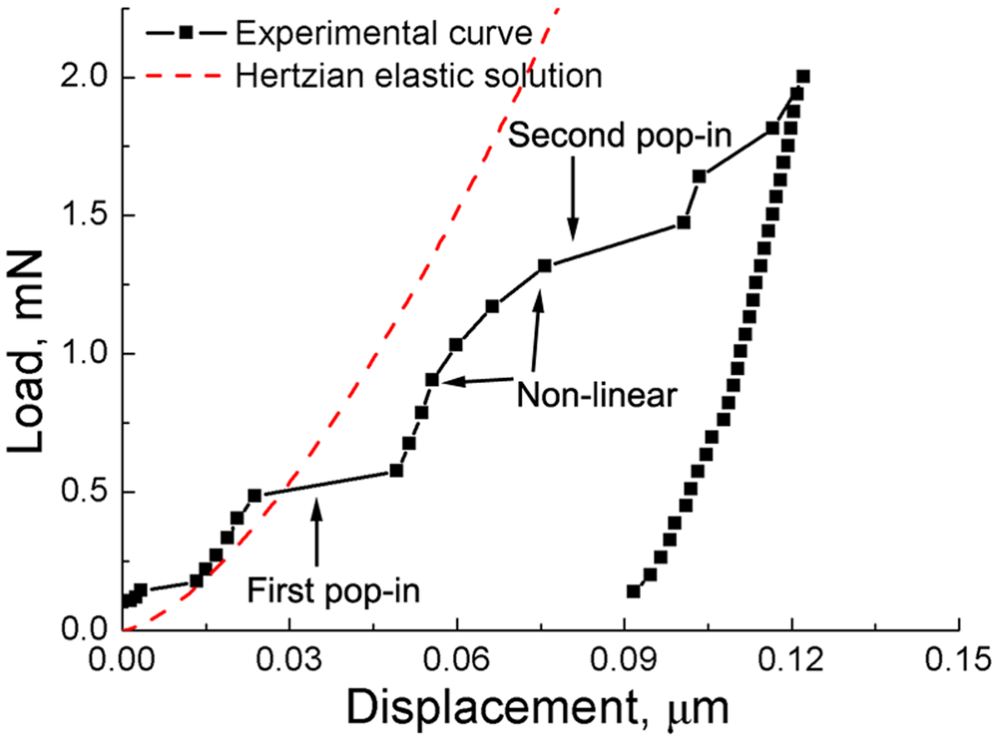
Load vs. displacement curve of the retained austenite indicated by the white square in Fig. 1, along with the Hertzian elastic solution.

Figure 2 shows the load-displacement curve of the RA indicated by the white square in Fig. 1. The pop-in like behaviour around 0.2 mN resulted from the initial contact of Berkovich diamond indenter with sample surface. The corresponding Hertzian elastic contact solution is as follows:^18^

1 $$P=\frac{4}{3}{E}_{r}{R}_{i}^{\frac{1}{2}}{h}^{\frac{3}{2}}$$

where *P* is the applied load, *h* is the corresponding depth of indentation, *R*_*i*_ is the radius of the indenter tip (160 nm in present study) and *E*_*r*_ is the effective Young’s modulus of indentation. For an isotropic elastic material, the effective indentation modulus *E*_*r*_ is given as:

2 $$\frac{1}{{E}_{r}}=\frac{1-{v}_{i}^{2}}{{E}_{i}^{2}}+\frac{1-{v}_{s}^{2}}{{E}_{s}^{2}}$$

where *E* is the Young’s modulus, *v* is the Poisson’s ratio, and the subscripts *i* and *s* represent the indenter and sample, respectively. For the indenter, *E*_*i*_ = 1141 GPa and *v*_*i*_ = 0.07^19^, whereas for austenite, *E*_*s*_ = 187 GPa and *v*_*s*_ = 0.3^20^. The Hertzian elastic solution was calculated following Eqs (1,2) and shown in Fig. 2. The load-displacement curve deviates from the Hertzian elastic solution at the first pop-in, which indicates the transition from elastic to plastic deformation. In the elastic regime, the maximum shear stress (*τ*_*m*_) is calculated as^18^:

3 $${{\rm{\tau }}}_{m}=0.31{(\frac{6P{E}_{r}^{2}}{{\pi }^{3}{R}_{i}^{2}})}^{1/3}$$

The initiation load of the first pop-in (0.48 mN) leads to a maximum shear stress of 16.0 GPa. This value is ~20% of the austenite shear modulus (76.8 GPa) at room temperature^21^, which is within the expected range for the theoretical yield strength of a crystalline material^22^. Thus, the first pop-in is likely due to the initiation of dislocation glide^11,12,19,23–25^.

**Figure 3:**
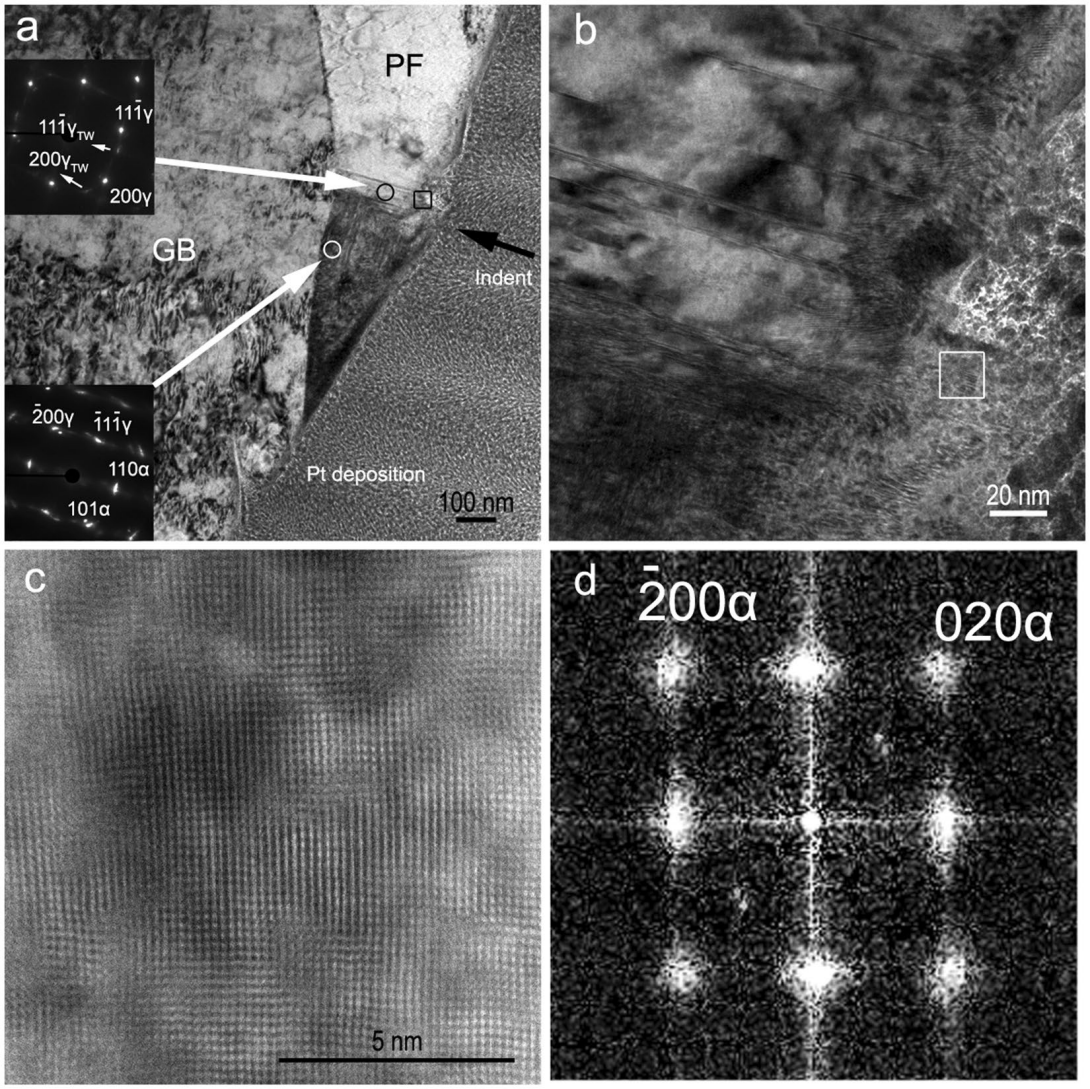
TEM images of cross-section of the indent area indicated by the white square in Fig. 1: (**a**) bright field image showing deformation twins (top-left SAD) and martensite formation (bottom-left SAD) in blocky RA adjacent to PF (the black arrow indicates the direction of indentation) with zone axes [011]*γ* and $[\bar{1}11]\alpha$ for austenite and martensite, respectively, (**b**) a zoomed-in view of the black square in (**a**) clearly showing deformation twins, (**c**) Wiener filtered annular bright image from the white square in (**b**) and (**d**) fast Fourier transform from (**c**) showing martensite at the [001]*α* zone axis. *RA* is retained austenite, *GB* is ferrite in granular bainite, and *PF* is polygonal ferrite.

Figure 3 shows the corresponding microstructures of the cross-section under the indent (Figs 1 (white square) and 2). As seen, the blocky RA in granular bainite partially twinned (upper-left selected area diffraction (SAD) in Fig. 3(a)) and partially transformed to martensite (lower-left SAD in Fig. 3(a) and fast Fourier transform in Fig. 3(d)). The SAD (lower-left inset) in Fig. 3(a) indicates Kurdjumov-Sachs (K-S) orientation relationship (OR) ({110}_bcc_//{111}_fcc_, 〈111〉_bcc_//〈101〉_fcc_) between the RA and martensite located far away from the indentation. On the other hand, Nishiyama-Wassermann (N-W) OR ({110}_bcc_//{111}_fcc_, 〈001〉_bcc_//〈101〉_fcc_) was observed between the RA (SADs in Fig. 3(a)) and martensite located just below the indentation (Figs 3(b, d)); in the more plastically deformed region. Both of these ORs were reported for TRIP steels^26^. In the present steel, only K-S OR was detected in previous studies^2,7^. The N-W OR observed here adjacent to the indentation may be due to the high local strain, leading to rotation from the K-S to N-W OR, as they only deviate from each other by 5.26°^26^.

As suggested in refs^11,12^, the second pop-in in the load-displacement curve (at a load of ~ 1.3 mN in Fig. 2) can be ascribed to martensite transformation. This is likely due to the volume expansion (~ 3% in iron-carbon alloys^27^) associated with the rapid diffusionless martensitic transformation process^11^. Here the observation of martensite under the indentation (Fig. 3) further supports the link between the second pop-in and martensite formation.

Of greater interest here is the non-linear part in the load-displacement curve (Fig. 2), as it has been seldom reported before. Based on the observed microstructures (Fig. 3), it is likely due to deformation twinning in RA. On one hand, contrary to martensitic transformation, deformation twinning in face-centred cubic (fcc) crystals is not associated with volume change^28^, and is a continuous process, not spontaneous like martensitic transformation. Thus, twinning is not expected to directly result in a pop-in. On the other hand, deformation twinning is associated with stress relaxation^28^, which in turn can lead to the observed deviation from linearity in Fig. 2.

**Figure 4:**
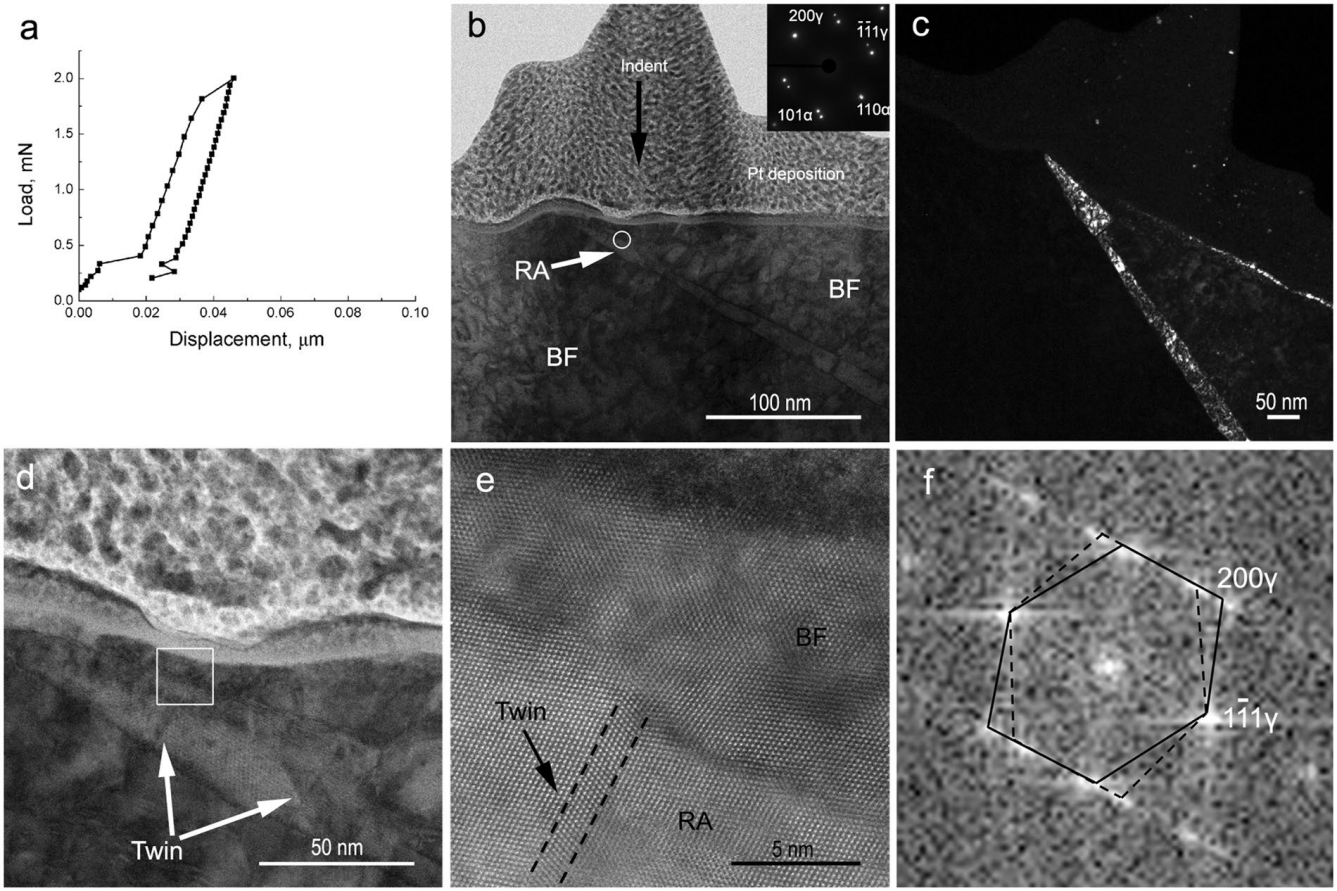
(**a**) Load vs displacement curve of the RA indicated by the white circle in Fig. 1 and (b–f) the corresponding TEM images of cross-section of the indent area: (**b**) bright field image showing film RA between BF laths (the black arrow indicates the direction of indentation), with zone axes [011]*γ* and $[\bar{1}11]\alpha$ for RA and BF lath, respectively, (**c**) dark field from (200)*γ*, (**d**) zoomed-in view of the white circle in (**b**); (**e**) high angle annular dark field STEM image from the white square in (**d**) showing RA, BF lath and deformation twin marked by dotted lines, and (**f**) fast Fourier transform from RA region showing the twin in (**e**). *RA* is retained austenite and *BF* is bainitic ferrite lath.

Figure 4(a) shows the load-displacement curve of the RA indicated by the white circle in Fig. 1. This area provides an example of deformation twinning in film RA between bainitic ferrite laths (Fig. 4(b,c)) characterised using annular bright field STEM imaging (Fig. 4(d,e)) together with fast Fourier transform (Fig. 4(f)). K-S OR between film RA and bainitic ferrite lath is shown via the SAD inset in Fig. 4(b). Compared to blocky RA in granular bainite (Fig. 3), no martensite was observed in this area, and accordingly the load-displacement curve (Fig. 4(a)) did not show the second pop-in associated with martensite transformation. Additionally, the non-linear part seen in Fig. 2 was not observed in Fig. 4(a); probably due to the formation of fewer deformation twins in this film RA. It is reiterated that the effect of the neighbouring bainitic ferrite laths on the load-displacement curve is unavoidable here due to the large applied load (2 mN) and the nano-sized film RA.

While deformation twinning typically occurs in low stacking fault energy austenitic steels, such as high manganese steels^29^, it has been rarely observed in RA in low-alloyed TRIP steel. Sugimoto *et al*.^15^. and Timokhina *et al*.^16^. reported deformation twinning in blocky RA after tensile loading of Fe-0.4C-1.5Si-1.5Mn-0.036Al (10% strain) and Fe-0.12C-1.77Si-1.39Mn-0.031Al-0.02Cr-0.005Cu (5% strain) TRIP steels, respectively. The present steel has similar chemical composition to refs^15,16^ and the observed deformation twining is probably due to the relatively high carbon content in RA (~ 6.0 at. %, as measured by atom probe tomography^7^) which significantly increases the stability of RA against martensitic transformation^10^.

The deformation twins divided the RA grains into sub-grains (Fig. 4(d)), then each sub-grain developed its deformation substructure by dislocation glide. Consequently, higher strain was required to initiate martensitic transformation and the overall RA stability was enhanced. When the strain is sufficient, the RA sub-grains would sequentially transform to martensite one by one if their volume is sufficiently large to initiate martensite nucleation (a minimum (sub)grain size of 0.7 µm was reported in ref.^5^). This behaviour was demonstrated by partial transformation of blocky RA to martensite (Fig. 3). The stepwise transformation manner may also contribute to the latter portion of the non-linear stage in the load-displacement curve (Fig. 2) as the small volume expansion associated with limited martensite formation would not be sufficient to result in a pop-in.

To verify the effect of twinning on RA stability, nine indents on blocky RA, which exhibited the second pop-in (indicating martensite transformation) but not the deviation from linearity (indicating no or very limited twinning), were used to estimate the onset load of martensite transformation and returned an average value of 0.93 ± 0.21 mN. However, when many twins were observed in blocky RA (Fig. 3), the onset load for martensite transformation was relatively larger (~1.3 mN in Fig. 2). These results underscore the enhancement of the overall RA stability by deformation twinning.

## Conclusion

Nanoindentation and correlative TEM observations of blocky and film RA grains indicate that the plastic deformation of RA occurs in the sequence of dislocation glide, twinning and martensitic transformation. When all these mechanisms are operative, they correspond to the first pop-in, non-linear part and second pop-in in the load-displacement curve, respectively. Deformation twinning enhanced the overall stability of RA via the division of RA grain into sub-grains. Lastly, the absence of martensitic transformation in film RA further supports its higher stability compared to the blocky RA.
